# RF-SVR-based prediction methodology for metal tube-bending rebound: Handling non-uniformity and limited sample challenges

**DOI:** 10.1371/journal.pone.0349240

**Published:** 2026-05-15

**Authors:** Ziluo Fang, Pengfei Zhang, Liangyou Li, Qingzhu Zhang

**Affiliations:** 1 Huzhou Key Laboratory of Intelligent Sensing and Optimal Control for Industrial Systems, School of Engineering, Huzhou Normal University, Huzhou, China; 2 Zhejiang Heliang Intelligent Equipment Co., Ltd., Huzhou, China; 3 School of Information and Control Engineering, China University of Mining and Technology, Xuzhou, China; University of Perugia: Universita degli Studi di Perugia, ITALY

## Abstract

This paper explores a prediction algorithm for determining the rebound angle of non-uniform and small-sample tubes. To address the issues of non-uniform and small-sample data, this paper proposes an algorithm based on Random Forest-Support Vector Regression (RF-SVR). Firstly, the polynomial feature generation method is introduced to solve the problem of non-uniform data. Secondly, after obtaining the data generated by the polynomial features, RF (Random Forest, an algorithm based on classification trees) is introduced to select the rebound features of the tubes, so that the features that have a profound influence on the rebound Angle can be retained. After obtaining the new data set, SVR (Support Vector Regression, an algorithm specifically designed for solving regression problems) is used to predict the rebound model of the bending of the metal tubes. The experimental results show that the RF-SVR method is superior to the traditional RF-BP and SVR methods, achieving higher prediction accuracy on small samples and non-uniform datasets.

## I. Introduction

**Fig 1:**
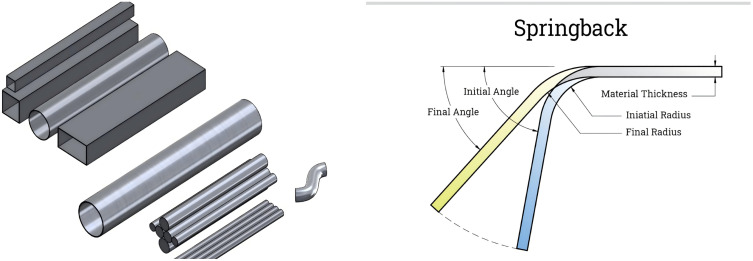
Illustration of industrial tubes and tube-bending rebound phenomenon. (a) common industrial metal tubes (b) tube-bending rebound (springback).

Metal tubes are used in various industrial sectors and applications due to their light weight, high strength, and excellent durability [1]. Pipeline layout often involves using tubes bending to address system requirements. CNC cold forming is now widely utilized to its energy efficiency, strong mechanical performance, and high production efficiency [2]. Compared with alternative lightweight materials such as PVC and fibreglass, steel tubes exhibit higher hardness and greater weight. These characteristics render the bending process more challenging and are accompanied by pronounced rebound phenomena. Rebound phenomenon is defined as the elastic recovery of the tube material after the removal of the external bending force, causing the bent tube to partially return toward its original straight configuration. This occurs due to the inhomogeneous deformation during bending: the outer wall experiences tensile strain while the inner wall undergoes compressive strain, generating residual stresses that are released upon unloading [3]. The industrial tubes and tube-bending rebound phenomenon are illustrated in Fig 1. The presence of rebound can disrupt the intended dimensions of the tubes, affecting its shape accuracy, as well as the structural installation and normal operation of the tubes [4]. By predicting the rebound of the tubes, the initial bending Angle can be pre-adjusted to achieve a size that meets the design specification. This approach helps prevent unnecessary rework and minimizes waste, especially for expensive materials. Therefore, rebound prediction plays a key role in improving bending quality, reducing costs, and improving manufacturing efficiency [3]. Thus, more and more research and attention has been paid to the prediction of rebound, with the focus on conducting tubes bending tests, analysing the influence of deformation conditions and tubes size on rebound, and implementing effective measurement, prediction and compensation strategies to ensure the precision and high quality of pipe bending manufacturing [5–7].

In practical industrial production, methods such as experimentation, analysis, or modeling are typically employed to predict and estimate tube rebound and compensation. Tang [8] researched tubes bending using maximum shear stress and plastic flow theory, analysing tubes deformation characteristics during bending. This study offers insights for predicting tube rebound through model analysis methods. Al-Qureshi [9] employed the coordinate deformation method to analysed axial and circumferential stress distribution during the bending of aluminium alloy tubes. It investigated the rebound radius ratio before and after unloading, residual stress post-unloading, and derived analytical formulas for tube diameter and symmetric bending sections. Zhan et al. [10] suggested employing numerical analysis to compute the rebound angle of thin-walled tubes, facilitating swift compensation for tube rebound. Based on model or analytical formula methods, the rebound issue of a specific material can be effectively addressed given known mechanical properties of the tubes. The experimental methods rely on extensive data from experiments conducted on the specific material to estimate rebound within certain intervals. While both methods predict rebound angles, the former requires comprehensive mechanical properties and specific parameters, whereas the latter demands ample experimental data on the material.

For many enterprises, complete mechanical property data for materials used in production or procurement are often unavailable and require specialized equipment for measurement. Moreover, most enterprises lack the equipment necessary to measure material mechanical properties. Furthermore, due to cost constraints and production limitations, conducting extensive bending experiments on specific materials to understand their bending characteristics is challenging for enterprises. Variations in performance among materials produced further complicate achieving accurate predictions. Hence, achieving rapid and precise rebound prediction and compensation across various materials remains an area requiring further exploration.

Data-driven approaches have shown remarkable effectiveness in several areas. The prediction of tubes rebound also belongs to the category of data prediction, and the data training method is also suitable for this field. Ma et al. [11] proposed a precise, efficient, and adaptable rebound control strategy using machine learning modeling. It developed an improved machine learning model based on the PSO-BP network to predict and compensate for the rebound of aluminium tubes. Compared with the traditional PSO-BP model, the crossover-operator model improves accuracy and reduces computation time from 1.5 h to 300 s. For rebound compensation, 32 iterations finish within 200 s, yielding results consistent with experimentsThe mean absolute error is 0.15°, relative error 10.49%, and maximum error 0.29°.Chen et al. [12] introduced a method for accurately predicting and efficiently controlling the rebound of tubes, considering various factors including material and geometric parameters. It successfully predicted and compensated for the rebound of alloy CNC bending tubes. Compared with the unoptimized BP network, the prediction accuracy improved by 18.5%.However, data training methods necessitate a substantial number of samples, making them susceptible to overfitting when dealing with limited data. This can lead to reduced prediction accuracy and suboptimal model performance. As a result, when faced with the challenge of limited rebound data for enterprise tubes, it becomes crucial to explore feasible alternative methods.

SVR is a regression method derived from support vector machines (SVM). It excels in handling small sample sizes, high-dimensional spaces, and nonlinear problems, making it effective for various predictive tasks. RF is an algorithm based on decision trees, particularly effective for complex classification tasks. It can analyse intricate interactions among features and rank them based on their importance, thereby enhancing model performance. Liu et al. [13] employed an experimental design method based on the I-optimal criterion to test four critical factors influencing forming accuracy. SVM was utilized to predict the rebound of W bending, yielding positive outcomes. SVR then used the most significant parameters of the steel structure as input, The results show that the predicted values closely match the experimental ones, with a minimum relative error of 0.3%. Chen et al. [14] conducted predictions and compensations for steel rebound, comparing the results with those obtained using a BP neural network. The findings demonstrated the superior performance of the support vector machine method. Baig et al. [15] collected the thickness, width, initial angle and other data of the steels, and established a prediction model based on random tree method to predict and compensate the rebound of the steels. Its mean absolute error is 0.41, and the mean squared error is 0.25. Because the tubes rebound prediction is a typical nonlinear regression problem, and there are many characteristics of tubes rebound, it is reasonable to select the more important characteristics for training. Therefore, for the tubes rebound prediction of small samples, SVR and RF are also feasible methods. In the actual production process of the enterprise, there may be non-uniform data, for example, some specific angle rebound data is missing or the properties of the tubes are difficult to obtain, which leads to the problem of non-uniform or missing data, thus affecting the training results of the model, so it is necessary to explore a feasible method to solve the problem of non-uniform rebound data of the tubes.

Feature generation is a part of feature engineering. Feature generation can increase feature expression ability and improve model effect. In the case of non-uniform or missing data, feature generation can improve data quality by filling in missing values through combination, interaction and clustering. The Markovitch et al. [16] proposes a framework that is capable of generating features from any given set of constructors by taking a set of classified objects, a set of attributes, and a set of constructor specifications containing their fields, ranges, and attributes as inputs, and generating a set of generated features as outputs. Hence, the framework is applied to various classification problems. Malik [17] proposes a space-based feature generation framework and applies it to machine learning processes. Finally, it is verified that the performance of machine learning techniques using space-based features is better. In the processing of tubes rebound data, the existing data information can be used to fill in the missing value through the method of feature generation, to solve the problem of missing and non-uniform data.

In this paper, RF-SVR is used for data processing and tubes rebound, and the proposed RF-SVR rebound algorithm has the following innovations:

1. **To solve the problem of small sample size, this paper introduces a method combining random forest feature ordering and SVR model training to effectively solve the influence of small sample size on model prediction.** Compared with the literature [8–12], RF is used for feature selection and SVR is used for prediction in this paper. When faced with lower data dimension and smaller capacity, RF-SVR shows better fitting performance than traditional model method or BP method, and the trained model also shows lower error.
2. **To solve the problem of non-uniform sample, this paper introduces the method of polynomial feature generation, which effectively solves the problem of data missing and non-uniform in the actual production process of enterprises.** Compared with the literature [13–15], polynomial feature generation is used to expand the tubes features in this paper. When the data is faced with the problem of missing or non-uniform features, the training effect of the data generated by polynomial features is better, and the prediction accuracy is higher after RF-SVR training.

In previous work, a GAN-SVR approach was proposed for predicting tube bending rebound with small samples. However, that method did not incorporate the mechanical properties of the tube material, which limited its physical interpretability and generalization capability. To overcome this limitation, the present study integrates key mechanical attributes of the tubes. Furthermore, guided by the physical characteristics of tube rebound, a novel feature generation strategy is introduced by incorporating coupled feature terms that reflect the interaction between geometric parameters and material properties. These enhancements improve the model accuracy and mechanistic relevance for rebound prediction.

## II. Problem statement and preliminaries

Section 2 introduces the research purpose and the basic structure of data processing model RF and prediction model SVR, finally describes the objective of this paper.

### A. Research purpose

In the actual production process of enterprises, the rebound of tubes is the key problem that affects the production efficiency and quality of tubes. Due to the diversity of the material and thickness of the tubes, the rebound phenomenon is difficult to be accurately calculated by a simple empirical formula. Consequently, it is necessary to study the rebound prediction of the bending tubes. By predicting and compensating the rebound of the tubes, the problem of the rebound of the tubes can be effectively solved. To accurately predict the rebound of the tubes, we must first collect the corresponding rebound data of the tubes as training, the tubes rebound data often has the problem of low dimension, and in the production process of practical enterprises, the tubes data for testing is generally less, and there is less data in some rebound interval or some tubes data missing. The problem of small samples and non-uniform or missing samples became two major obstacles. For one thing, high-quality experimental data are often difficult to obtain in copious quantities. On the other hand, even if a certain amount of data is collected, the distribution of these data in various parameter dimensions may be non-uniform or there may be some data missing. To sum up, it is important to solve these problems through appropriate models to improve the performance of predictive models. In this paper, data training models RF and SVR are adopted to solve the above problems.

### B. Random forest

**Fig 2:**
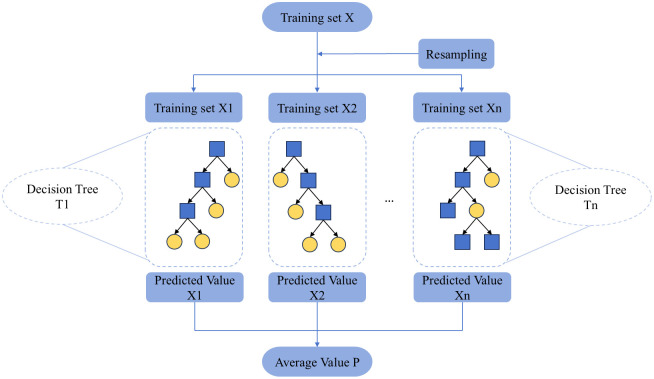
The structure of random forests.

Random forest (RF) is an algorithm based on classification trees [18]. RF utilizes random resampling techniques to extract multiple samples from the original dataset and constructs numerous decision trees through random node splitting. By aggregating the predictions of these trees via voting, RF generates the final classification result. This method is particularly effective in analysing classification features and is robust against noisy data and missing values. Additionally, RF is known for its rapid learning speed. The variable importance measurement provided by RF serves as a valuable feature selection tool, especially for high-dimensional datasets. Over recent years, RF has found widespread application in diverse areas including classification, prediction, feature selection, and outlier detection problems [19]. The structure of RF is shown in Fig 2.

As can be seen from the structure of RF, RF consists of multiple decision trees, and the final forecast results are summarized by voting or averaging. Secondly, assuming that there are n features in the original data, m features m≤n are randomly selected from each node of each tree. Through calculation, a feature with the strongest classification ability is selected for node splitting.

The ensemble of trees in RF is used to classify new data. The classification outcome is determined by aggregating the predictions of each individual tree classifier. The final classification decision can be summarized as follows:


$$\begin{array}{c} \begin{array}{c} H(x)=arg\underset{Y}{\text{max}}\sum_{i=\text{1}}^{k}I(h_{i}(x)=Y) \end{array} \end{array}$$ (1)

Where H(x) represents a combinatorial classification model, $h_{i}$ represents a single decision tree classification model, *Y* represents an output variable, and I(·) represents a real function.

### C. Support vector regression

**Fig 3:**
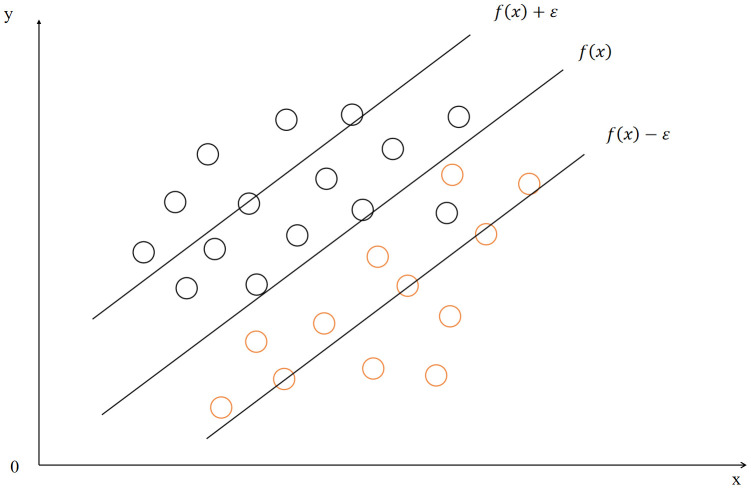
The structure of SVM.

Support vector regression (SVR) is an algorithm rooted in support vector machines, specifically designed for solving regression problems. SVR aims to model data by maximizing the margin between predictions and actual values, while permitting a specified degree of error tolerance. Unlike traditional regression approaches, SVR excels in handling nonlinear relationships and high-dimensional data [20]. The support vector regression is shown in Fig 3.

The main idea of SVM is to find an optimal hyperplane that separates the different classes of data points and maximizes the distance of the boundary from the closest point of the two classes of data points.

Given the training samples set $\left(x_{i},y_{i}\right),i=\text{1},\text{2},\cdots ,l,x\in R^{n},y\in \{\pm \text{1}\}$, the hyperplane is (ω·x)+b=0. To classify all samples correctly and have classification intervals, it is required to satisfy the relation as follows:


$$\begin{array}{c} \begin{array}{c} y_{i}\left[\left(\omega \cdot x_{i}\right)+b\right]\geq \text{1},i=\text{1},\text{2},\cdots ,l \end{array} \end{array}$$ (2)

It follows that the classification interval is 2/‖ω‖, so the problem of constructing the optimal hyperplane is transformed into the constrained minimum problem as follows:


$$\begin{array}{c} \begin{array}{c} min\Phi (\omega )=\frac{\text{1}}{\text{2}}{\left\|\omega \right\|}^{\text{2}}=\frac{\text{1}}{\text{2}}{(\omega }^{\prime }\cdot \omega ) \end{array} \end{array}$$ (3)

Introduce the Lagrange function as follows:


$$\begin{array}{c} \begin{array}{c} L=\frac{\text{1}}{\text{2}}{\left\|\omega \right\|}^{\text{2}}-\sum_{i=\text{1}}^{l}a_{i}y_{i}\left(\omega \cdot x_{i}+b\right)+\sum_{i=\text{1}}^{l}a_{i},a_{i}>\text{0} \end{array} \end{array}$$ (4)

Where $a_{i}$ is the Lagrange coefficient. The QP problem is transformed into the corresponding dual problem as follows:


$$\begin{array}{c} \begin{array}{c} maxQ(a)=\sum_{j=\text{1}}^{l}a_{j}-\frac{\text{1}}{\text{2}}\sum_{i=\text{1}}^{l}\sum_{j=\text{1}}^{l}a_{i}a_{j}y_{i}y_{j}\left(x_{i}x_{j}\right),\\ a_{j}>\text{0},j=\text{1},\text{2},\cdots ,l \end{array} \end{array}$$ (5)

The optimal weight vector $\omega^{*}$ and the optimal bias $b^{*}$ can be obtained by calculation as follows:


$$\begin{array}{c} \begin{array}{c} \omega^{*}=\sum_{j=\text{1}}^{l}\overset{*}{\underset{j}{a}}y_{j}x_{j} \end{array} \end{array}$$ (6)

$$\begin{array}{c} \begin{array}{c} b^{*}=y_{i}-\sum_{j=\text{1}}^{l}y_{j}\overset{*}{\underset{j}{a}}(x_{j}\cdot x_{i}) \end{array} \end{array}$$ (7)

Therefore, the optimal classification surface $(\omega^{*}\cdot x)+b^{*}=\text{0}$ is obtained, and the optimal classification function is shown as follows:


$$\begin{array}{c} \begin{array}{c} f(x)=sgn\left\{\left(\omega^{*}\cdot x\right)+b^{*}\right\},x\in R^{n} \end{array} \end{array}$$ (8)

where $\omega^{*}$ is the weight vector and $b^{*}$ is the optimal bias.

### D. Objective

In the process of tubes rebound prediction and compensation, small samples and non-uniform tubes data are important problems affecting tubes rebound prediction, so the objectives of this paper are as follows:

(1) Introducing a method to realize parameters and characteristics of the homogenization, alleviate non-uniform samples of the effects on the rebound prediction.
(2) Introducing a method to alleviate the training bias caused by small samples and improve the prediction accuracy of the training model.

## III. Prediction algorithm of tubes rebound based on RF-SVR conclusion

Section 3 mainly introduces the RF-SVR prediction method for small non-uniform sample tubes data. By this method, the rebound of tubes can be effectively predicted based on some known mechanical properties of bending tubes. The RF-SVR tubes rebound prediction algorithm proposed in this paper is shown in Table 1 as follows.

**Table 1:** Prediction algorithm of tubes rebound based on RF-SVR.

Step 1 Data preprocessing	This step includes data cleaning, data conversion, data normalization, and data partitioning.According to scaling formula: $\overset{*}{\underset{i}{x}}=\frac{x_{i}-\underset{i\leq j\leq n}{min}\left\{x_{j}\right\}}{\underset{i\leq j\leq n}{max}\left\{x_{j}\right\}-\underset{i\leq j\leq n}{min}\left\{x_{j}\right\}}$,the data is normalized and divided into trainingset and test set according to requirements.
Step 2 Feature polynomial generation	This step is based on the original parameters, according to the following formula: $f(x)=a_{\text{0}}+a_{\text{1}}x_{\text{1}}+\cdots +a_{n}x_{n}+a_{n+\text{1}}x_{\text{1}}x_{\text{2}}+\cdots +a_{m}x_{n-\text{1}}x_{n}+a_{m+\text{1}}\overset{\text{2}}{\underset{\text{1}}{x}}+\cdots +a_{l}\overset{\text{2}}{\underset{n}{x}}$, each parameter is power combined or feature cross to produce a new feature.
Step 3 Feature selection based on RF	This step is based on the parameters generated after the feature; the parameter is used as the input of RF for feature selection. Among the many parameters, according to the formula of importance: ${VIM}_{j}=\sum_{i=\text{1}}^{n}{VIM}_{ij},$ screen out the main features that affect the target variable.
Step 4 Prediction of SVR	In this step, the data after feature selection is used as the input of SVR to obtain the prediction curve of tubes rebound. Finally, the test set is used to evaluate the tubes rebound curve and improve the prediction accuracy through iterative optimization.

### A. Data preprocessing

**Fig 4:**
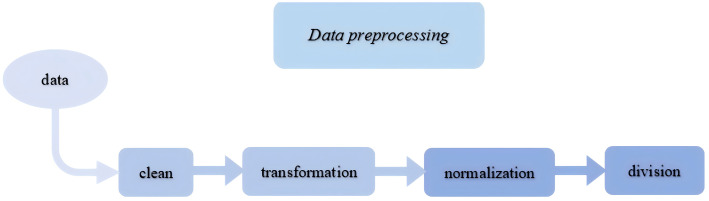
The flow chart of data processing.

In this paper, the data was collected from a cooperative intelligent manufacturing enterprise of tubes bending, and a total of 150 sets of data were collected for three types of tubes (copper, aluminium and stainless steel). Each type of tubes collected a rebound data every 10° from 10° to 100°, and a total of fifty sets of data for each type of tubes. The attribute parameters of the tubes are selected from the commonly available open parameters in the literature. Before data training, the data should be pre-processed. In the pre-processing stage, the original data is cleaned, transformed, normalized and re-divided to eliminate noise and outliers in the data, extract effective features and reduce the complexity of the data. The specific process is shown in the Fig 4.

(1) Data cleaning is one of the core steps of preprocessing. By detecting and correcting errors or duplicate values in the data, we can ensure an accurate and reliable data set.
(2) Data transformation is an operation to adapt data to a predictive model. This may include converting non-numerical data into numerical data that can be understood and processed by the model. At the same time, feature selection or new features can be created to better reflect the relevance and complexity of the data.
(3) Data normalization is to eliminate dimensional differences between data and ensure that unique features have similar scales. This helps to avoid overfitting problems by focusing the model too much on certain data.
(4) Data division is to prepare for subsequent data processing. To verify the accuracy of the algorithm, two materials are used as the experimental group in the tubes data collected, and the remaining material is used as the prediction group. In addition, for each material of the metal tubes rebound data, also according to the material properties, rebound Angle and rebound Angle are divided.

### B. Feature polynomial generation

Feature generation can capture the coupling effect between material properties. The coupling terms of some mechanical properties of tubes (such as elastic modulus and yield strength) have a influence on the rebound behavior [21]. The introduction of such interaction terms not only has clear physical meaning, but also significantly improves the interpretability of the model.

For small sample data, the simplified quadratic term model can improve its predictive ability [22]. For a primitive feature {x,y}, the primitive feature can be extended by a quadratic polynomial as shown as follows:


$$\begin{array}{c} \begin{array}{c} f(x,y)=\text{1}+x+y+xy+x^{\text{2}}+y^{\text{2}} \end{array} \end{array}$$ (9)

Through the above transformation of quadratic polynomials, the original feature {x,y} is transformed into a new feature set {$\text{1},x,y,xy,x^{\text{2}},y^{\text{2}}$}. For the case of non-uniform tubes data, the generation of polynomial features for tubes data features can use existing data information to fill in the missing values, and the generated polynomial features can fully consider the distribution and correlation of existing data, to make up for the problem of non-uniform data to a certain extent.

The tubes data collected in this paper include attribute parameters and rebound parameters, among which attribute parameters include inner diameter, wall thickness, elastic modulus, yield strength, tensile strength, elongation; rebound parameters include bending angle and rebound angle. The process of polynomial feature generation is to generate the power combination of each parameter or cross the features based on the original parameters, to get a new set of features. All the attribute parameters and the bending Angle in the rebound parameters constitute the input of the rebound of the tubes, the input parameters are set as $\left\{x_{\text{1}},x_{\text{2}},x_{\text{3}},x_{\text{4}},x_{\text{5}},x_{\text{6}},x_{\text{7}}\right\}$. Set the degree of the polynomial to two and the number of cross-terms to two, augmented by the quadratic polynomial as shown as follows:


$$\begin{array}{c} \begin{array}{c} f(x)=\text{1}+x_{\text{1}}+\cdots +x_{\text{7}}+x_{\text{1}}x_{\text{2}}+\cdots +x_{\text{6}}x_{\text{7}}+\overset{\text{2}}{\underset{\text{1}}{x}}+\cdots +\overset{\text{2}}{\underset{\text{7}}{x}} \end{array} \end{array}$$ (10)

By extension, the 36-dimensional input can be obtained as follows:


$$\begin{array}{c} \begin{array}{c} \left\{\text{1},x_{\text{1}},\cdots ,x_{\text{7}},x_{\text{1}}x_{\text{2}},\cdots ,x_{\text{6}}x_{\text{7}},\overset{\text{2}}{\underset{\text{1}}{x}},\cdots ,\overset{\text{2}}{\underset{\text{7}}{x}}\right\} \end{array} \end{array}$$ (11)

When dealing with the problem of bending tubes rebound with small samples, quadratic polynomial generation is usually chosen, because quadratic polynomial generation can effectively prevent overfitting and adapt to small sample data. And because the feature operations involved in quadratic polynomial generation are simple, it is easier to understand how the model fits the data.

### C. Feature selection based on RF

RF-based feature selection is a widely used method that employs Random Forest models to assess the significance of each feature concerning the predicted target. This technique identifies features that significantly influence model performance for subsequent analysis or modeling. When handling limited sample sizes, it becomes crucial to prioritize essential features. Pre-modeling feature selection enables capturing correlations and nonlinear associations between features effectively. This approach adeptly models intricate feature relationships inherent in small sample datasets while mitigating redundancy by excluding highly correlated features. Random forests can effectively avoid overfitting when dealing with small sample data [23]. RF-based feature selection methods encompass the following approaches:

(1) Mean Decrease in Impurity: The importance of a feature is assessed by calculating the degree to which each feature is used to reduce impurity in all decision trees.
(2) Permutation importance method: The importance of a feature is evaluated by randomly shuffling the value of a feature and observing the degree of impact on model performance.
(3) SHAP value method: SHAP value is used to explain the process of model prediction, to evaluate the contribution of each feature to model prediction.

In this paper, the average impurity decrease (i.e., average Gini impurity) is used to measure the contribution degree of each feature to the model performance, to carry out feature selection.

The generated tubes samples are $\left(x_{i},y\right)$, the number of samples is *D*, i=1,2,3⋯, where $x_{i}$ represents the input parameter of the 36-dimension tubes rebound, and *y* represents the output parameter of the tubes rebound. When calculating the importance of the feature parameter $x_{i}$, a decision tree *i* is taken as the starting node, and the Gini value of $x_{i}$ in the *i* th decision tree is calculated as follows:


$$\begin{array}{c} \begin{array}{c} {GI}_{j}(x_{i})=\sum_{i=\text{1}}^{D}P^{\text{2}}\left(\frac{y}{x_{i}}\right)-\text{1} \end{array} \end{array}$$ (12)

By calculating the GI value corresponding to each decision tree in RF and taking the average value, we can get the importance of the feature information $x_{i}$.


$$\begin{array}{c} \begin{array}{c} IMP(x_{i})=\frac{\text{1}}{N}\sum_{j=\text{1}}^{N}{GI}_{j}(x_{i}) \end{array} \end{array}$$ (13)

### D. Prediction of tubes rebound based on RF-SVR

**Fig 5:**
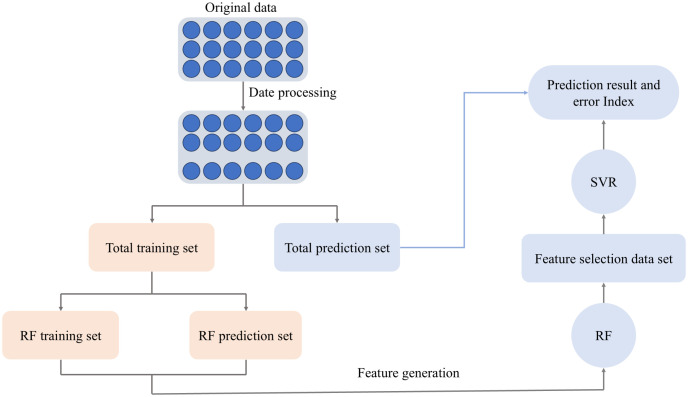
The structure of Prediction of tubes rebound based on RF-SVR.

The structure of Prediction of tubes rebound based on RF-SVR is shown in the Fig 5 as follows.

For the small sample problem of tubes rebound, SVR can well alleviate the influence of small samples on model prediction, and the features selected by feature selection as the input of SVR can avoid the influence of invalid features on the model, to improve the prediction accuracy. The literatures [24] proposes that the rebound data of bending tubes has strong nonlinear characteristics. Linear kernel is suitable for linear sample data, and polynomial check is not concerned with points near the sample, while RBF kernel is more concerned with sample points near the support vector [25,26]. Therefore, the data type studied in this paper should choose RBF core. The specific form is as follows.


$$\begin{array}{c} \begin{array}{c} K\left(x_{i},x\right)=\text{exp}\left(-\frac{{\left\|x_{i}-x\right\|}^{\text{2}}}{\text{2}\sigma^{\text{2}}}\right) \end{array} \end{array}$$ (14)

## IV. Experimental setup and data collection

**Fig 6:**
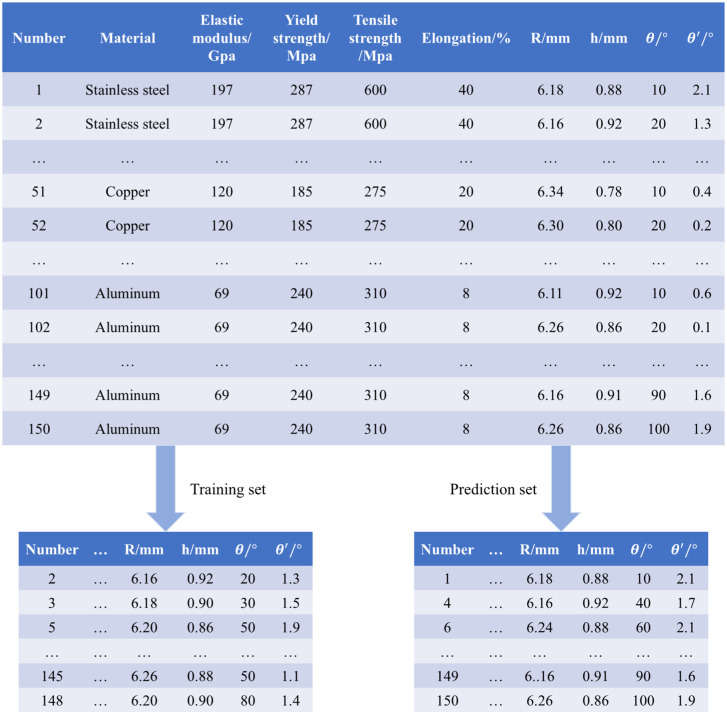
Tubes rebound data set.

The total set data was mixed and randomly divided into training set and prediction set according to the ratio of 2:1, as shown in the Fig 6 as follows.

Due to the significant differences between parameter values, data normalization should be processed before data training. Commonly used data normalization methods include maximum and minimum value normalization method, logarithmic function normalization method and vector normalization method. In this paper, the maximum-minimum normalization method is adopted to scale the data according to the following formula:


$$\begin{array}{c} \begin{array}{c} \overset{*}{\underset{i}{x}}=\frac{x_{i}-\underset{i\leq j\leq n}{min}\left\{x_{j}\right\}}{\underset{i\leq j\leq n}{max}\left\{x_{j}\right\}-\underset{i\leq j\leq n}{min}\left\{x_{j}\right\}} \end{array} \end{array}$$ (15)

The state x represents the parameters before normalization, the state $x^{*}$ represents the parameters after normalization.

**Fig 7:**
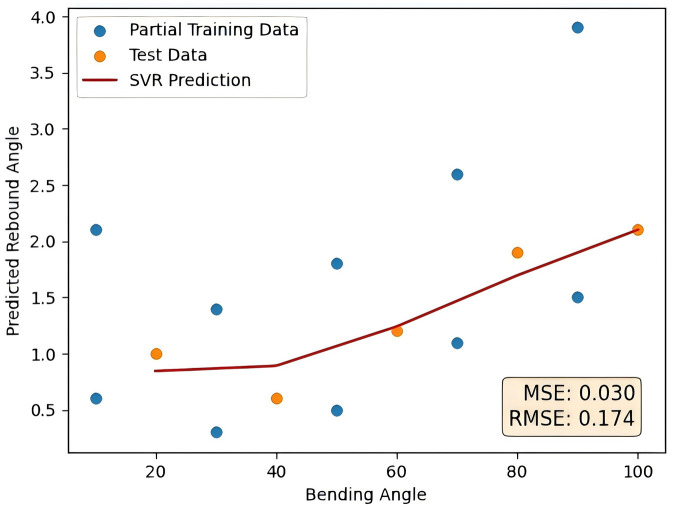
RF-SVR prediction results.

**Fig 8:**
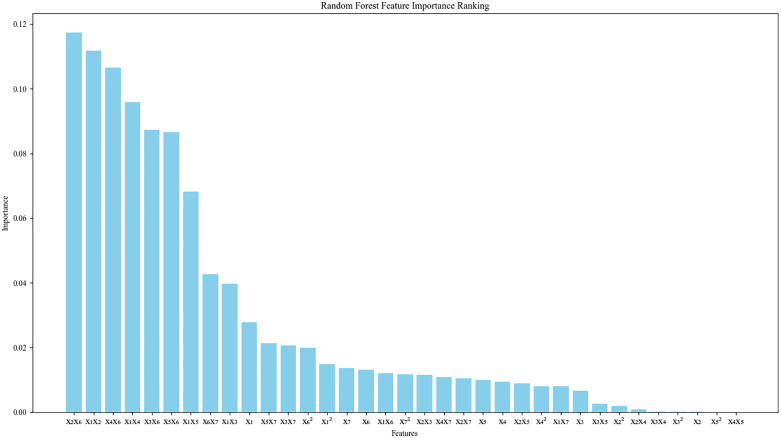
Random forest importance ranking.

To eliminate the impact of non-uniform and missing data on the model, feature generation is performed on the data first, and the data is generated using quadratic polynomial feature generation. The generated data is shown in Table 2. The RF training set and RF prediction set data after feature generation are used as input of the random forest for feature selection. Finally, the feature parameters after feature selection are retained. After screening, a new total training set is obtained as the input of SVR, Bayesian optimization is used to optimize the hyperparameters of RF and SVR, including the number of decision trees, the maximum depth of the decision tree, the minimum number of samples required for decision tree splitting, regularization parameters, kernel function parameters, and error tolerance, and the prediction curve of tubes rebound is obtained. The prediction curve is shown in the Fig 7. The ranking of feature importance is shown in Fig 8.

**Table 2:** The data after feature generation.

Number	$x_{1}$	$x_{2}$	…	$x_{7}$	$x_{1}x_{2}$	$x_{1}x_{3}$	…	$x_{6}x_{7}$	$\overset{2}{\underset{1}{x}}$	$\overset{2}{\underset{2}{x}}$	…	<<Eqn51>>	c
1	197	287	…	10	56539	118200	…	8.8	38809	82369	…	100	1
2	197	287	…	20	56539	118200	…	18.4	38809	82369	…	400	1
…	…	…	…	…	…	…	…	…	…	…	…	…	…
149	69	240	…	90	16560	21390	…	81.9	4761	57600	…	8100	1
150	69	240	…	100	16560	21390	…	86	4761	57600	…	10000	1

This figure shows the prediction results of the RF-SVR method proposed in this paper. In this paper, MSE and RMSE are used as evaluation indicators, and the specific form is shown as follows.


$$\begin{array}{c} MSE=\frac{\text{1}}{m}\sum_{i=\text{1}}^{m}{\left(y_{i}-{\hat{y}}_{i}\right)}^{\text{2}} \end{array}$$ (16)

$$\begin{array}{c} RMSE=\sqrt{\frac{\text{1}}{m}\sum_{i=\text{1}}^{m}{\left(y_{i}-{\hat{y}}_{i}\right)}^{\text{2}}} \end{array}$$ (17)

## V. Experimental results and analysis

**Fig 9:**
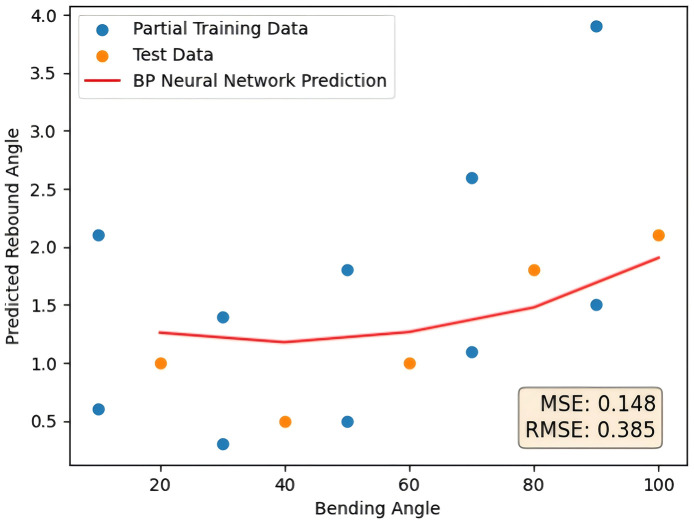
RF-BP prediction results.

As a contrast, this paper takes BP neural network and SVR as a comparison to verify the adaptability of the SVR method proposed in this paper to small sample data. The feature parameters of the random forest after feature selection are also used as the input of the BP neural network and its prediction curve is shown in Fig 9 as follows. The error comparison with the method proposed in this paper is shown in Table 3.

**Table 3:** Comparison of RF-BP and RF-SVR results.

Prediction method	RF-BP	RF-SVR
MSE	0.148	0.030
RMSE	0.385	0.174

Compared with the RF-BP model, the RF-SVR model reduces the MSE by approximately 80% and the RMSE by approximately 55%. As shown in the prediction curves, the RF-SVR model demonstrates superior accuracy and exhibits stronger capability in handling small-sample problems in tube rebound prediction.

**Fig 10:**
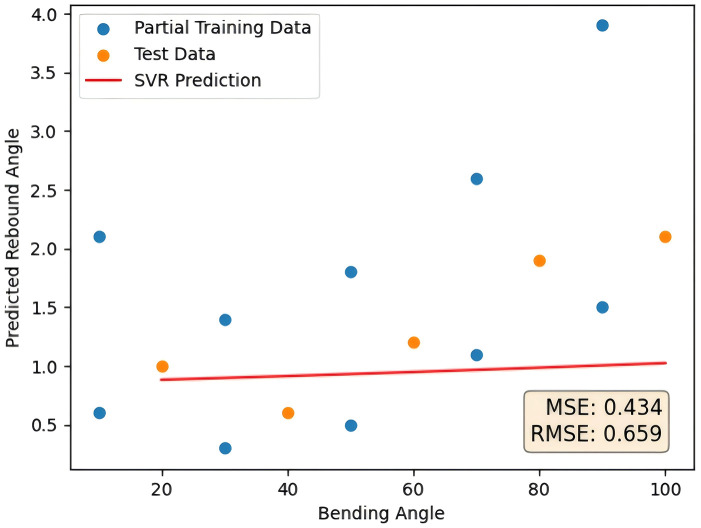
SVR prediction results.

As a contrast, this paper directly uses SVR and RF-SVR for comparison, divides the original data into prediction set and test set, and carries out normalization processing. The prediction set is directly used as the input of SVR, and the test set is used as the performance evaluation. The prediction curve is shown in Fig 10 as follows. The error comparison with the method proposed in this paper is shown in Table 4.

**Table 4:** Comparison of SVR and RF-SVR results.

Prediction method	SVR	RF-SVR
MSE	0.434	0.030
RMSE	0.659	0.174

Compared with the SVR model, the RF-SVR model reduces the MSE by approximately 93% and the RMSE by approximately 73%. As shown in the prediction curves, the RF-SVR model demonstrates superior accuracy and exhibits stronger capability in handling small-sample problems in tube rebound prediction.

**Fig 11:**
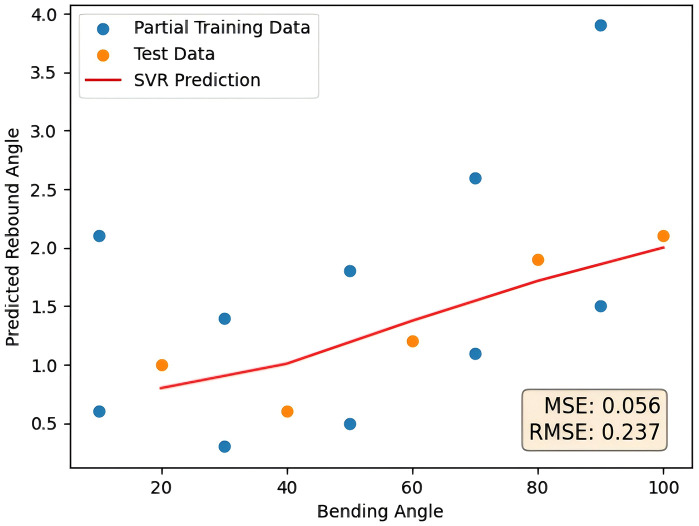
Non-uniform data prediction results.

As a contrast, the simulated missing data set is compared with the complete data set to verify the benefits of the polynomial feature generation method proposed in this paper for data inhomogeneity or missing data. Removing a number of data for a material from the initial tubes rebound data in the experiment, and then the same steps were carried out. The final prediction curve was shown in Fig 11 as follows. The error comparison with the method proposed in this paper is shown in Table 5.

**Table 5:** Comparison of non-uniform data and complete data results.

Prediction method	Non-uniform data	Complete data
MSE	0.056	0.030
RMSE	0.237	0.174

Compared with complete data, the prediction using non‑uniform data shows only slightly higher errors, with MSE increasing from 0.030 to 0.056 and RMSE from 0.174 to 0.237. The results remain within an acceptable range, demonstrating strong robustness and anti‑interference capability of the method.

**Fig 12:**
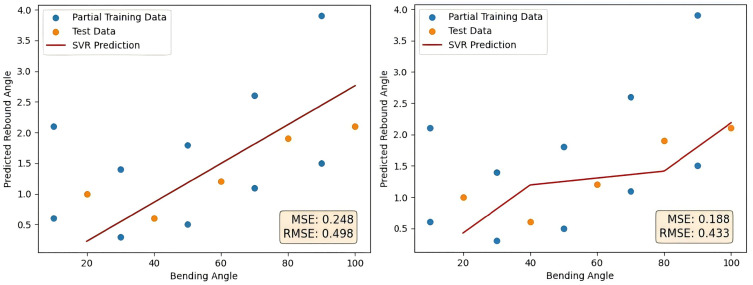
Linear kernel and polynomial kernel prediction results.

As a contrast, linear kernel, polynomial kernel and Gaussian kernel are used to verify the processing capability of the proposed Gaussian kernel in SVR squadron small sample data. The above experiment is repeated by changing the kernel function of SVR to linear kernel and polynomial kernel. The final forecast curve is shown in Fig 12 as follows. The error comparison with the method proposed in this paper is shown in Table 6.

**Table 6:** Comparison of linear kernel data, polynomial kernel and RBF kernel results.

Prediction method	Linear kernel	Polynomial kernel	RBF kernel
MSE	0.248	0.188	0.030
RMSE	0.498	0.433	0.174

Among the three kernels, the RBF kernel achieves the best performance, MSE and RMSE representing reductions of about 88% and 65% compared to the linear kernel, and 84% and 60% compared to the polynomial kernel. These results highlight the superior accuracy and robustness of the RBF kernel in prediction tasks.

**Fig 13:**
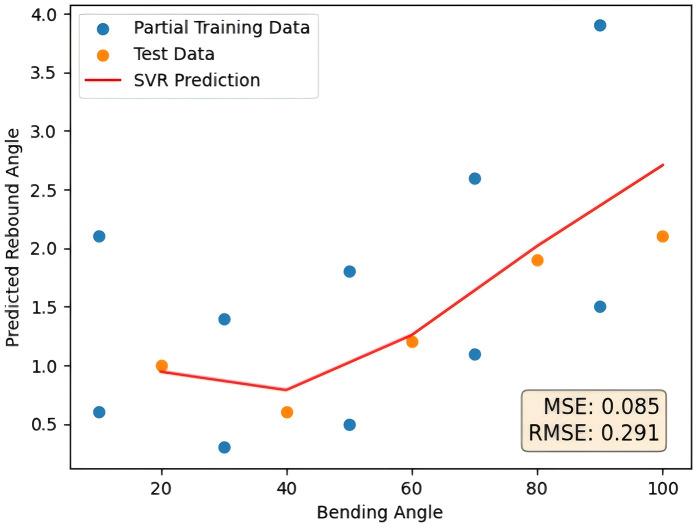
SHAP method and replacement importance method prediction results.

As a contrast, SHAP value method, displacement importance method and average impurity reduction method are used as comparison in this paper to verify the advantages of the average impurity reduction method as a random forest feature selection method and processing small sample data of tubes rebound. The feature selection method of RF was changed to SHAP value method and replacement importance method. The remaining steps did not change, and the final prediction curve was shown in Fig 13 as follows. The error comparison with the method proposed in this paper is shown in Table 7.

**Table 7:** Comparison of SHAP method, replacement importance method and Mean Decrease in Impurity method kernel results.

Prediction method	SHAP method	Replacement importance method	Mean Decrease in Impurity method
MSE	0.085	0.130	0.030
RMSE	0.291	0.433	0.174

Among the three importance methods, the Mean Decrease achieves the best performance, reducing errors by about 65% and 77% compared to the SHAP and replacement importance methods. This demonstrates its superior accuracy in prediction tasks.

**Fig 14:**
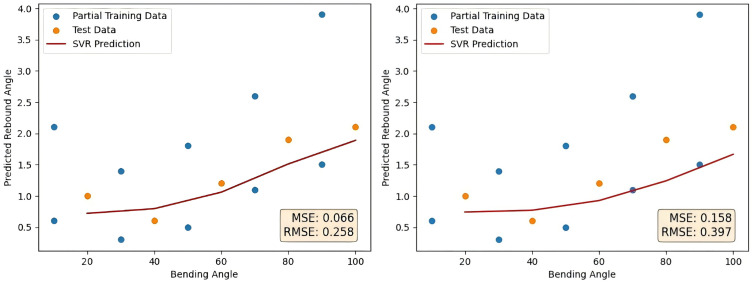
Degrees 3 and degrees 4 prediction results.

As a contrast, the degree of polynomial generation is set to 3 and 4, which verifies the advantage of selecting quadratic polynomial generation for small sample data of tubes rebound. Change the degree of polynomial generation to 3 and 4. The remaining steps do not change, and the final prediction curve is shown in Fig 14 as follows. The error comparison with the method proposed in this paper is shown in Table 8.

**Table 8:** Comparison of degrees 3, degrees 4 and degrees 2 results.

Prediction method	Degrees 3	Degrees 4	Degrees 2
MSE	0.066	0.158	0.030
RMSE	0.258	0.397	0.174

Among the polynomial degrees tested, degree 2 achieves the best performance. Compared with degree 3, this represents reductions of about 55% in MSE and 33% in RMSE, while compared with degree 4, the reductions are about 81% and 56%, respectively. These results highlight the superior accuracy of the degree 2 model in prediction tasks.

**Fig 15:**
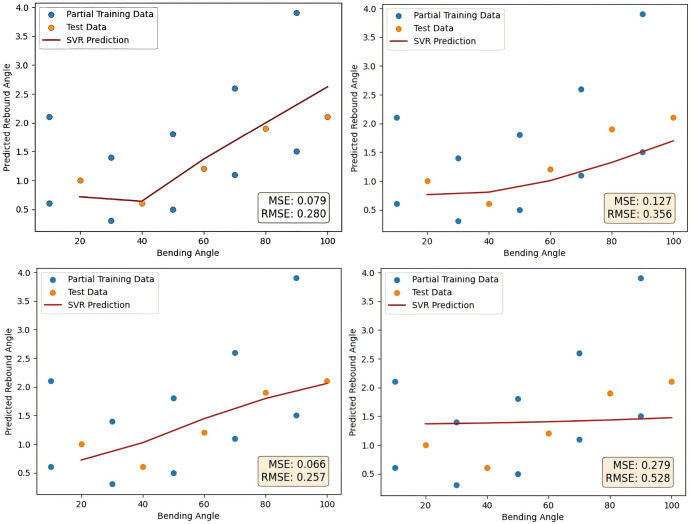
Four normalization methods prediction results.

As a contrast, Z-score normalization method, decimal scaling normalization method, logarithmic function normalization method and vector normalization method are used to verify the advantages of maximum and minimum value normalization method as a data normalization method to deal with small sample data of tubes rebound. The normalization method of the data is changed to the above four methods, the other steps remain unchanged, and the final prediction curve is shown in Fig 15 as follows.The error comparison with the method proposed in this paper is shown in Table 9.

**Table 9:** Comparison of the results of five normalization methods.

Prediction method	Z-score normalization method	Decimal scaling normalization method	
MSE	0.079	0.127	
RMSE	0.280	0.356	
**Prediction method**	**Logarithmic function normalization method**	**Vector normalization method**	**Maximum and minimum value normalization method**
MSE	0.066	0.279	0.030
RMSE	0.257	0.528	0.174

Compared with the other four normalization methods, the maximum-minimum normalization method shows clear advantages, it reduces MSE by about 62% compared with Z-score, 76% compared with decimal scaling, 55% compared with logarithmic function, and 89% compared with vector normalization. For RMSE, the reductions are about 38%, 51%, 32%, and 67%, respectively. These results highlight its superior accuracy in prediction tasks.

**Fig 16:**
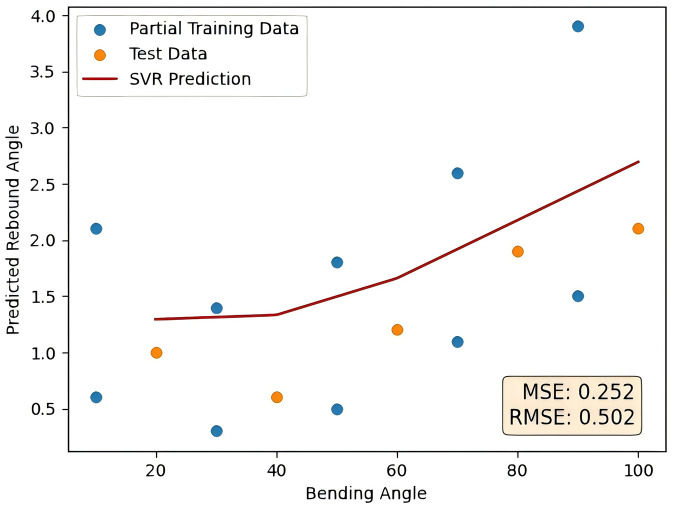
Trigonometric function generation method prediction results.

As a contrast, the function used for feature generation was changed to a trigonometric function, verifying the advantage of quadratic polynomial generation in processing tube rebound sample data. The prediction curve generated by the trigonometric function generation method is shown in Fig 16 as follows. The error comparison with the method proposed in this paper is shown in Table 10.

**Table 10:** Comparison of the results of trigonometric function generation and quadratic polynomial generation.

Prediction method	Trigonometric function generation	Quadratic polynomial generation
MSE	0.252	0.030
RMSE	0.502	0.174

Compared with the trigonometric function generation, the quadratic polynomial approach performs significantly better, reducing MSE by about 88% and RMSE by about 65% compared with the trigonometric function method. This highlights its superior accuracy in prediction tasks.

**Fig 17:**
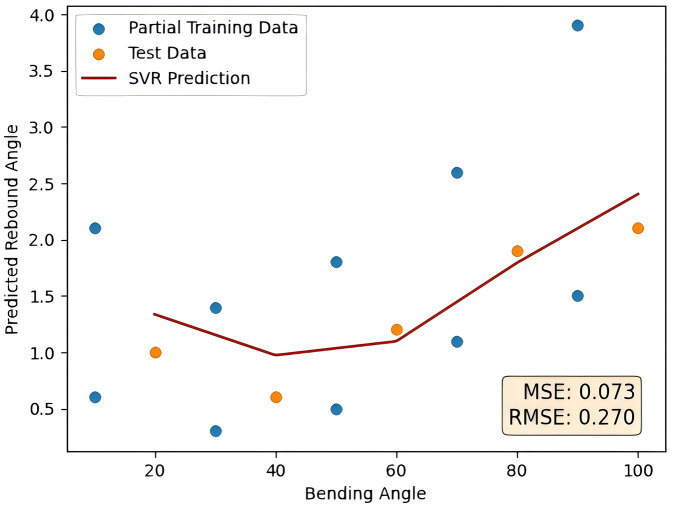
Adaptive boosting method prediction results.

As a contrast, the adaptive boosting method was used to rank the importance of features, verifying the advantages of random forest feature selection in processing tubes rebound data. The prediction curve using the adaptive boosting algorithm is shown in Fig 17 as follows. The error comparison with the method proposed in this paper is shown in Table 11.

**Table 11:** Comparison of the results of adaptive boosting method and random forest feature selection.

Prediction method	Adaptive boosting method	Random forest feature selection
MSE	0.073	0.030
RMSE	0.270	0.174

Compared with the adaptive boosting method, random forest feature selection performs better, reducing MSE by about 59% and RMSE by about 36% compared with adaptive boosting. This demonstrates its superior accuracy in prediction tasks.

## VI. Conclusions

**Fig 18:**
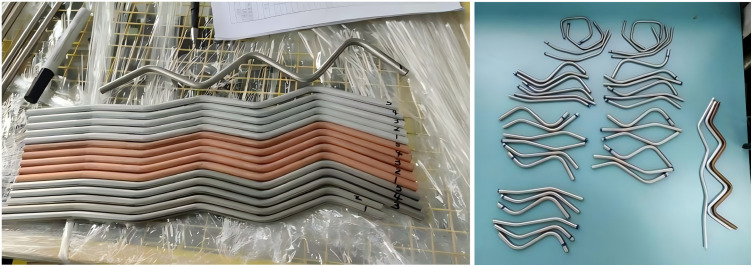
Representative examples of industrial tubes.

This paper is an innovative expansion of team previous paper [27]. This paper presents an RF-SVR algorithm for predicting the rebound angle of small sample and non-uniform tubes. Compared with SVR and RF-BP, RF-SVR achieves higher accuracy, with prediction errors reduced by more than 70% in RMSE and over 80% in MSE. Specifically, the RF-SVR model attains MSE is 0.030 and RMSE is 0.174. Experimental validation further demonstrates the effectiveness of quadratic polynomial generation, maximum-minimum normalization, Gaussian kernel selection, and Mean Decrease in Impurity, each contributing error reductions ranging from 30% to 90% relative to alternative methods.These findings provide a reliable approach for processing and predicting tube bending rebound data and open new directions for future research (Fig 18).
